# NBM-T-BBX-OS01, Semisynthesized from Osthole, Induced G1 Growth Arrest through HDAC6 Inhibition in Lung Cancer Cells

**DOI:** 10.3390/molecules20058000

**Published:** 2015-05-04

**Authors:** Jih-Tung Pai, Chia-Yun Hsu, Kuo-Tai Hua, Sheng-Yung Yu, Chung-Yang Huang, Chia-Nan Chen, Chiung-Ho Liao, Meng-Shih Weng

**Affiliations:** 1 Division of Hematology and Oncology, Tao-Yuan General Hospital, Ministry of Health and Welfare, Taoyuan City 33004, Taiwan; E-Mail: jihtungpai@gmail.com; 2 Department of Nutritional Science, Fu Jen Catholic University, New Taipei City 24205, Taiwan; E-Mails: sandy0805w@hotmail.com (C.-Y.H.); j06103@hotmail.com (S.-Y.Y.); 3 Graduate Institute of Toxicology, College of Medicine, National Taiwan University, Taipei 10051, Taiwan; E-Mail: d94447003@gmail.com; 4 NatureWise Biotech and Medicals Corporation, Taipei 11559, Taiwan; E-Mails: naturewisecyh@gmail.com (C.-Y.H.); alex_chen@gnt.com.tw (C.-N.C.); 5 Division of Drug and New Technology Product, Food and Drug Administration, Ministry of Health and Welfare, Executive Yuan, Taipei 11516, Taiwan; E-Mail: cedar@fda.gov.tw

**Keywords:** histone deacetylase, heat shock protein 90, cell cycle arrest, NBM-T-BBX-OS01 (TBBX), suberoylanilide hydroxamic acid (SAHA), lung cancer

## Abstract

Disrupting lung tumor growth via histone deacetylases (HDACs) inhibition is a strategy for cancer therapy or prevention. Targeting HDAC6 may disturb the maturation of heat shock protein 90 (Hsp90) mediated cell cycle regulation. In this study, we demonstrated the effects of semisynthesized NBM-T-BBX-OS01 (TBBX) from osthole on HDAC6-mediated growth arrest in lung cancer cells. The results exhibited that the anti-proliferative activity of TBBX in numerous lung cancer cells was more potent than suberoylanilide hydroxamic acid (SAHA), a clinically approved pan-HDAC inhibitor, and the growth inhibitory effect has been mediated through G1 growth arrest. Furthermore, the protein levels of cyclin D1, CDK2 and CDK4 were reduced while cyclin E and CDK inhibitor, p21^Waf1/Cip1^, were up-regulated in TBBX-treated H1299 cells. The results also displayed that TBBX inhibited HDAC6 activity via down-regulation HDAC6 protein expression. TBBX induced Hsp90 hyper-acetylation and led to the disruption of cyclin D1/Hsp90 and CDK4/Hsp90 association following the degradation of cyclin D1 and CDK4 proteins through proteasome. Ectopic expression of HDAC6 rescued TBBX-induced G1 arrest in H1299 cells. Conclusively, the data suggested that TBBX induced G1 growth arrest may mediate HDAC6-caused Hsp90 hyper-acetylation and consequently increased the degradation of cyclin D1 and CDK4.

## 1. Introduction

Lung cancers are the leading cause of cancer mortality and threat to health in the world [1]. Multiple genetic and epigenetic alternations are involved in lung tumorigenesis [2]. Those alternations carry worse prognosis and result in resistance to chemotherapy and radiation [3,4]. Aberrant epigenetic modulation of gene expression has been indicated as an important mechanism in tumorigenesis. Epigenetics is traditionally defined as the study of heritable changes in gene expression caused by mechanisms other than changes in the underlying DNA sequence. Emerging evidence indicates that the epigenome is dynamic and changes in responses to many factors, such as environment, diet, disease and aging [5]. Furthermore, epigenetic changes are demonstrated as a partial cause of the chemoresistance [6]. Therefore, modulations of histone acetylation status have been verified as therapeutic and/or chemopreventive targets of cancer [7]. Suberoylanilide hydroxamic acid (SAHA), the first histone deacetylases (HDACs) inhibitor, has been certified to inhibit many cancer cell proliferations and, further, has been approved by Food and Drug Administration for the treatment of cutaneous manifestations of T-cell lymphoma [8]. Therefore, targeting epigenetic regulators is a strategy for cancer therapy. Previous studies identified that HDAC6 displayed as a better target than other HDACs because of its high relevant cancer-related non-histone substrates without the severe toxicity [9,10]. Thus, HDAC6 might provide a new target for cancer treatment.

The modification of histone acetylation via histone acetyltransferase (HAT)/histone deacetylase (HDAC) system is an important post-translational modification mechanism in directing gene expression. HATs transfer acetyl group from acetyl-CoA molecule to lysine of histone and gene expression is then turned on. Conversely, HDACs remove the acetyl group from histone, leading to the repression of gene expression by stabilizing DNA-histone interaction [11]. Non-histone proteins are also confirmed as HDAC substrates, which rule cell proliferation, survival and differentiation [12,13,14,15,16,17]. Eighteen HDACs have been discovered and are divided into class I, II, III and IV [18]. Class I HDACs, which are located in the nucleus and modulated histone acetylation, include HDAC1, 2, 3, and 8. Class IIa HDAC family consists of 4, 5, 7 and 9, whereas isoforms 6 and 10 are members of class IIb HDAC [19]. Recent evidence indicates that targeting HDACs has been shown to induce cell cycle disruption in tumor cell models [20,21]. HDAC1 has been revealed to have over-expression and correlation with poor prognosis in lung cancer patients [22,23]. The inhibition of class I HDAC activity induces growth arrest and apoptosis by inducing p21^Waf1/Cip1^ gene expression in tumor cell [24,25,26]. Down-regulation of CDK1, 2, and 4 protein expression, resulting in cell cycle arrest at G1 phase via HDAC inhibitor, has been observed [27]. Otherwise, class IIb HDAC6 is also recognized as an oncogene [28,29]. HDAC6 primarily deacetylates non-histone substrates, such as -tubulin, cortactin and heat-shock protein 90 (Hsp90), ensuing microtubule stabilization and microtubule-mediated processes [30,31]. Meanwhile, HDAC6 regulates protein stability via changing acetylation status of Hsp90 by the repression of Hsp90 chaperonee complexes [14,16,32]. Serial client proteins, such as epidermal growth factor receptor (EGFR), glucocorticoid receptor, vascular endothelial growth factor receptor, mutant p53 and cyclin-dependent kinases (CDKs), have been shown to complex with Hsp90. The disruption of Hsp90 chaperone-function leads to client protein degradation following apoptosis and/or growth arrest in cancer cells [15,32,33,34,35].

Cell cycle is an important process of cell proliferation, growth and cell division. Disruption of the normal regulation of cell cycle progression and division are critical events in the development of cancer, including in lung cancer. Malignant lung cells possess the ability to pass cell cycle checkpoints, which are associated with aberrant expression of cell cycle regulators, such as cyclins and CDKs [36,37]. Highly expression of cyclin D1 protein has been displayed in invasive lung cancer cells [38], and is correlated with low survival rate and poor prognosis of lung cancer patients [39]. D-type cyclins and their binding kinases, CDKs, direct cell cycle G1-S transition [36]. Down-regulation of D-type cyclin expression and cyclin-CDK activities are detected during growth factor deprivation following arrest cell at G1 phase [40,41]. Obstruction of D-type cyclins expression and cyclin-CDK activities via small molecules manipulating proliferation inhibition is strategy for cancer treatment. Recently, down-regulation of cyclin D via HDAC6 inhibition to block cell proliferation has been verified in lung and breast cancer cells [29,42]. However, the relationship between cell cycle arrest and HDAC6 inhibition in lung cancer is still unclear. 

**Figure 1:**
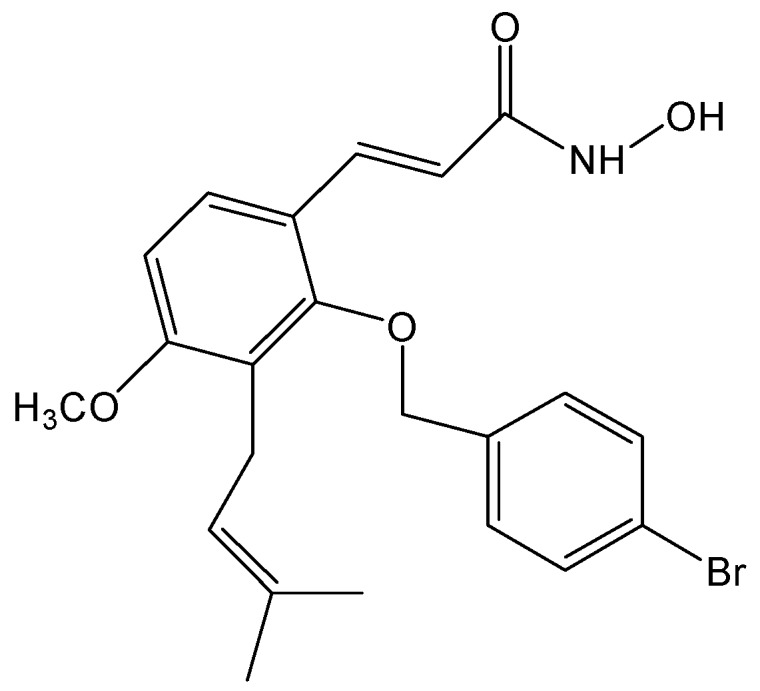
Structure of NBM-T-BBX-OS01 (TBBX).

Most of HDAC inhibitors are classified as pan-inhibitors, such as SAHA and FK228 [43]. Targeting individual HDAC family member has high specificity and low toxicity benefits. Recently, a derivative semisynthesized from ostholes, NBM-T-BMX-OS01, has been identified as a potent HDAC8 inhibitor and enhances learning and memory in rats [44]. In the present study, anti-proliferative effect of NBM-T-BBX-OS01 (TBBX) (Figure 1) was further investigated in lung cancer cells. TBBX was also derived from osthole and structurally as an analog of NBM-T-BMX-OS01. Our data exhibited that TBBX was a HDAC6 inhibitor via down-regulation HDAC6 expression. TBBX induced G1 cell cycle arrest might be through down-regulation HDAC6 expression and followed by hyper-acetylation of Hsp90 and accelerating cyclin D1 and CDK4 degradation in H1299 lung cancer cells.

## 2. Results and Discussion

### 2.1. TBBX Induced G1 Growth Arrest in Human Lung Cancer Cells

**Figure 2:**
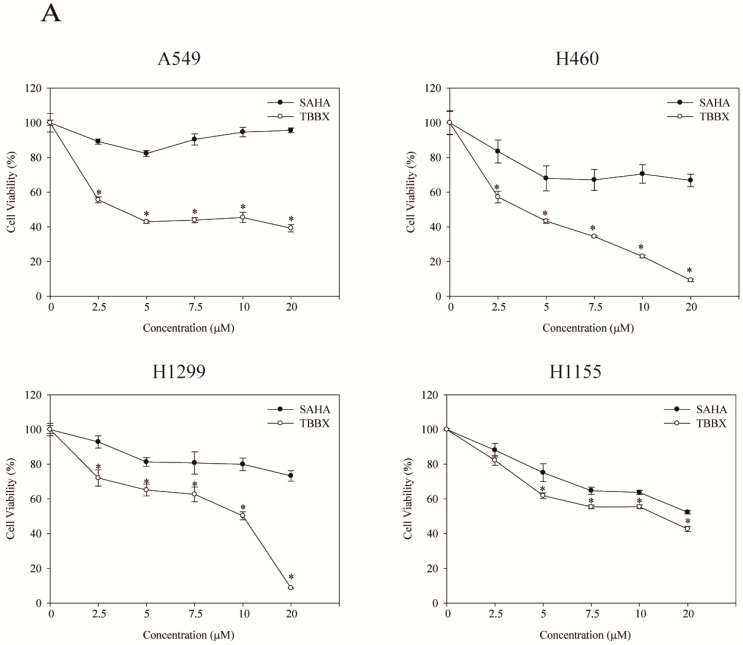

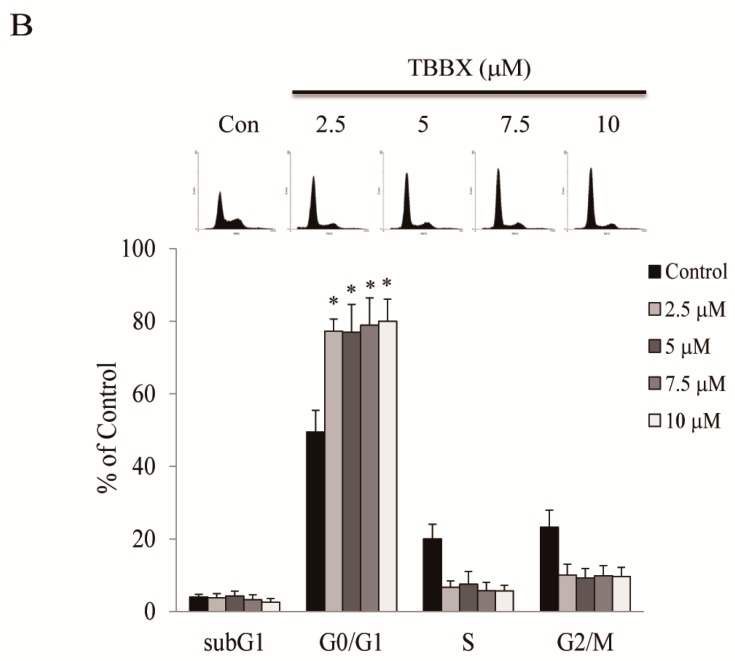
The growth inhibition effect of TBBX in H1299 lung cancer cells. (**A**) A549, H1299, H460 and H1155 cells were incubated with a serial dosage (0, 2.5, 5, 7.5, 10 and 20 μM) of TBBX or suberoylanilide hydroxamic acid (SAHA) for 24 h. Afterward, MTT assay was performed for cell viability as described in Materials and Methods. Data shown are representative of at least three independent experiments. (**B**) H1299 cells were seeded in a 10 cm petri dish for 24 h. Cells were then treated with various doses of TBBX for 24 h. After cells were harvested, flow cytomertic analysis was performed for cell cycle distribution. Data were the mean ± S.D. of triplicate samples. Significant difference was observed from the control group (* *p* < 0.05).

The inhibitory effect of TBBX in numerous lung cells has been examined through cell viability assay. Cells were treated with the serial dosages of TBBX (0, 2.5, 5, 7.5, 10 μM) for 24 h. The cell viability was then measured by MTT assay. As shown in Figure 2A, the growth inhibitory effect of TBBX was observed in dose-dependent manners. The anti-proliferative activity of TBBX in lung cancer was more potent than SAHA (Figure 2A). To further evaluate whether the growth inhibitory effect of TBBX was through cell cycle disruption, H1299 cell line was chosen as the model. H1299 cells were treated with various concentrations of TBBX for 24 h and flow cytomerty was executed for cell cycle analyses. Figure 2B showed that up to 30% of G1 phase (from 49.5% to 79.9%) was boosted after 10 μM TBBX treatment in H1299 cells. The results revealed that TBBX significantly induced G1 cell cycle arrest in H1299 cells.

**Figure 3:**
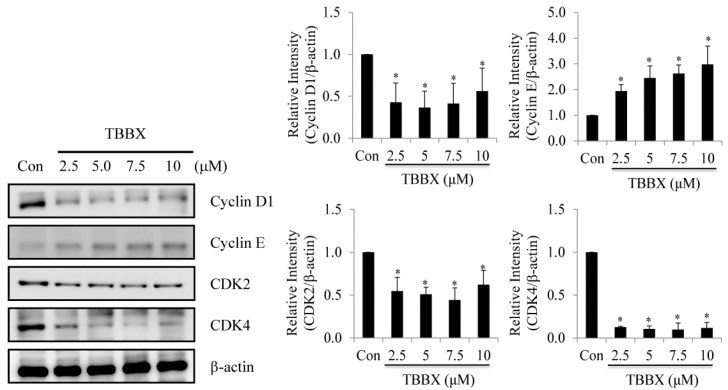
Effects of TBBX on the expressions of cyclins, and CDKs in H1299 lung cancer cells. H1299 lung cancer cells were initially synchronized by serum-free medium and then serum-supplemented medium containing various doses of TBBX (0, 2.5, 5, 7.5, and 10 μM). After the cells were harvested, Western blot analyses were performed with anti-cyclin D1, E, CDK2, CDK4, and β-actin antibodies. Data shown are representative of at least three independent experiments. Significant difference was observed from the control group (* *p* < 0.05).

In order to further investigate molecular mechanism of TBBX-induced cell cycle arrest, H1299 cells were synchronized and treated with TBBX. After 24 h treatment, cells were harvested and the expression of cyclin D1, E, CDK2 and CDK4 were inspected by Western blotting. The protein levels of cyclin D1, CDK2 and CDK4 were decreased with TBBX treatment, while the expressions of cyclin E was increased (Figure 3).

### 2.2. Up-Regulation of CDK Inhibitors Was Observed in TBBX-Treated H1299 Lung Cancer Cells

**Figure 4:**
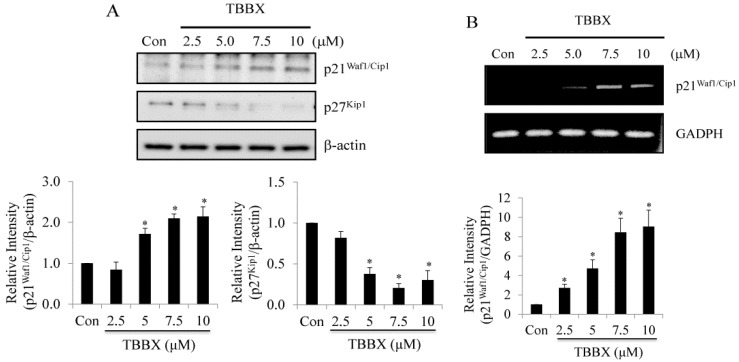
Effects of TBBX on the expression of CDK inhibitors, p21^Waf1/Cip1^ and p27^Kip1^, in lung carcinoma H1299 cells. H1299 lung cancer cells were initially synchronized by serum-free medium and then serum-supplemented medium containing various doses of TBBX (0, 2.5, 5, 7.5, and 10 μM) for 24 h. After the cells were harvested, (**A**) Western blot analyses were performed with anti-p21^Waf1/Cip1^, p27^Kip1^ and anti-β-actin antibodies. (**B**) H1299 cells were treated with TBBX for 12 h and total mRNAs were extracted afterward. After the extraction of total mRNAs, p21^Waf1/Cip1^ and GAPDH RT-PCR were performed as described in Materials and Methods. Data shown are representative of at least three independent experiments. Significant difference was observed from the control group (* *p* < 0.05).

It has been well characterized that CDK activity is inhibited by CDK inhibitors, p21^Waf1/Cip1^ and p27^Kip1^. The complex activities of cyclins/CDKs associated with p21^Waf1/Cip1^ and p27^Kip1^ were repressed resulting in cell cycle arrest [45,46]. Therefore, the effects of TBBX on the expression of p21^Waf1/Cip1^ and p27^Kip1^ were characterized by Western blot (Figure 4A). The protein levels of p21^Waf1/Cip1^ were up-regulated via TBBX in a dose-dependent mode. However, the expression of p27^Kip1^ was decreased in TBBX-treated cells (Figure 4A). To further investigate the mechanism of TBBX-induced p21^Cip1/Waf1^ expression, H1299 cells were treated with TBBX for 12 h. Total RNAs were collected and RT-PCR was then performed. The results confirmed that p21^Waf1/Cip1^ mRNA expression was increased in a dose-dependent manner (Figure 4B). The outcomes implicated that TBBX induced G1 cell cycle arrest might be through up-regulated the protein level of p21^Waf1/Cip1^ rather than p27^Kip1^ expression. Up-regulation of p21^Waf1/Cip1^ expression was through transcriptional regulation.

### 2.3. Class I HDACs Were Not Involved in TBBX-Induced Growth Arrest in H1299 Lung Cancer Cells

**Figure 5:**
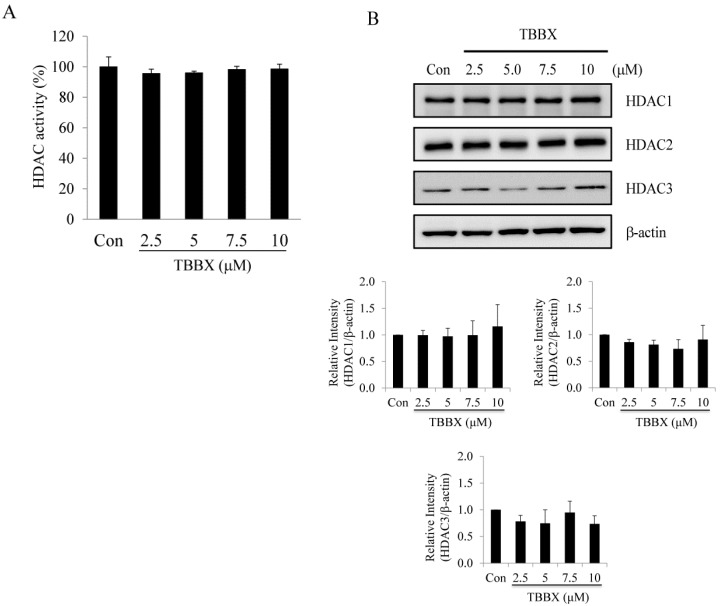
Effects of TBBX on the class I HDAC activity and protein expression in H1299 lung cancer cells. (**A**) Direct inhibition class I HDAC activity assay by TBBX was performed as described in Materials and Methods. (**B**) H1299 cells were treated with various dosage of TBBX (0, 2.5, 5, 7.5, and 10 μM) for 24 h. After treatment, cells were harvested and Western blot was done by anti-HDAC1, HDAC2, HDAC3 and anti-β-actin antibodies. Data shown are representative of at least three independent experiments.

It has been demonstrated that down-regulation HDAC activity gives rise to G1 cell cycle arrest via inducing p21^Waf1/Cip1^ expression [24,25]. To determine whether p21^Waf1/Cip1^-mediated growth arrest by TBBX treatment was through HDACs inhibition, class I HDAC activity assay was directly performed by cell-free system. As shown in Figure 5A, class I HDAC activity was not affected with TBBX treatment. TBBX-treated H1299 cell lysates were harvested for HDAC 1, 2 and 3 protein expression analyses to further study the effects of TBBX on class I HDAC expression. The data revealed that the protein levels of HDAC 1, 2 and 3 were not altered between control and TBBX-treated H1299 cells (Figure 5B).

### 2.4. TBBX-Prompted Cyclin D1 and CDK4 Degradation Was through Interruption of Hsp90 with Cyclin D1 and CDK4 Association

**Figure 6:**
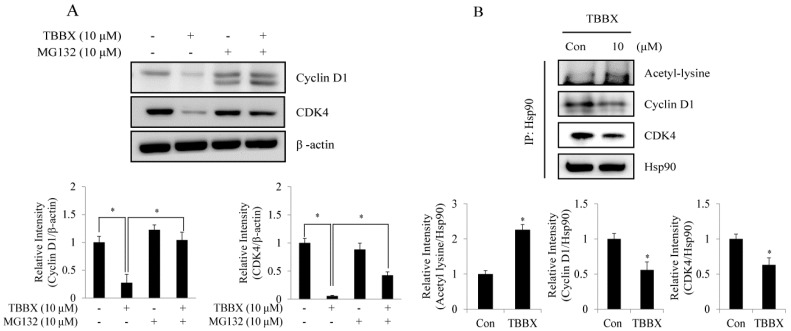
TBBX induced Hsp90 hyper-acetylation and disrupted cyclin D1/Hsp90 and CDK4/Hsp90 interaction and leading to cyclin D1 and CDK4 degradation in H1299 lung cancer cells. (**A**) H1299 cells were incubated with or without 10 μM MG132 for 30 min before 10 μM TBBX stimulation for 24 h. Cells were harvested to detect cyclin D1 and CDK4 expression by Western blotting analyses. (**B**) H1299 cells were incubated with 10 μM TBBX for 24 h. Cells were then harvested and immuno-precipitation analysis was down with anti-Hsp90 antibody. The immuno-precipitates were then detected by Western blotting with anti-cyclin D1, anti-CDK4 and anti-acetyl-lysine antibodies as described in Materials and Methods. Data shown are representative of at least three independent experiments. Significant difference was observed from the control group (* *p* < 0.05).

Disruption of Hsp90 chaperone function is well known to suppress cell cycle progression through promoting cell cycle regulator degradation by proteasome system [14,15]. To understand the down-regulation mechanism of cyclin D1 and CDK4 in TBBX-stimulated cells, H1299 cells were pretreated with proteasome inhibitor MG132 for 30 min before TBBX treatment. As shown in Figure 6A, TBBX-down-regulated CDK4 and cyclin D1 expression was rescued by MG132 pretreatment to validate the proteasome degradation involved in treated cells. Additionally, immune-precipitation of Hsp90 was performed to identify the roles of cyclin D1/Hsp90 and CDK4/Hsp90 association in the function of Hsp90 chaperon in TBBX-down-regulated cyclin D1 and CDK4 expression. The results unveiled that the bound protein levels of cyclin D1 and CDK4 with Hsp90 were significantly decreased about 40% in TBBX-treated cells (Figure 6B). The outcomes implicated that TBBX-down-regulated cyclin D1 and CDK4 expression might be through disrupting interaction with Hsp90.

### 2.5. TBBX-Induced G1 Growth Arrest Was Mediated by HDAC6-Regulated Hsp90

**Figure 7:**
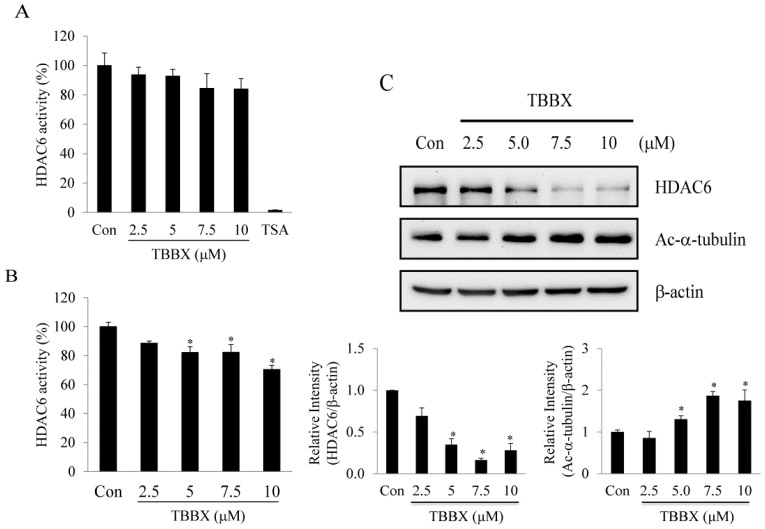
Effect of TBBX on class IIb HDAC6 activity and protein expression in H1299 lung cancer cells. (**A**) Nuclear extracts containing HDAC6 enzymes were incubated with various concentrations of TBBX (0, 2.5, 5, 7.5 and 10 μM) and HDAC6 activity assay was determined as described in Materials and Methods. The trichostatin A (TSA, 2 μM) were examined as positive control. (**B**) H1299 cells were incubated with various dosage of TBBX (0, 2.5, 5, 7.5 and 10 μM) for 24 h. Cells were harvested and HDAC6 activity was determined as described in Material and Methods. (**C**) H1299 cells were treated with various dosage of TBBX (0, 2.5, 5, 7.5, and 10 μM) for 24 h. After treatment, cells were harvested and Western blot was done by anti-HDAC6, acetyl-tubulin and anti-β-actin antibodies. Data were the mean ± S.D. of triplicate samples. Significant difference was observed from the control group (* *p* < 0.05).

**Figure 8:**
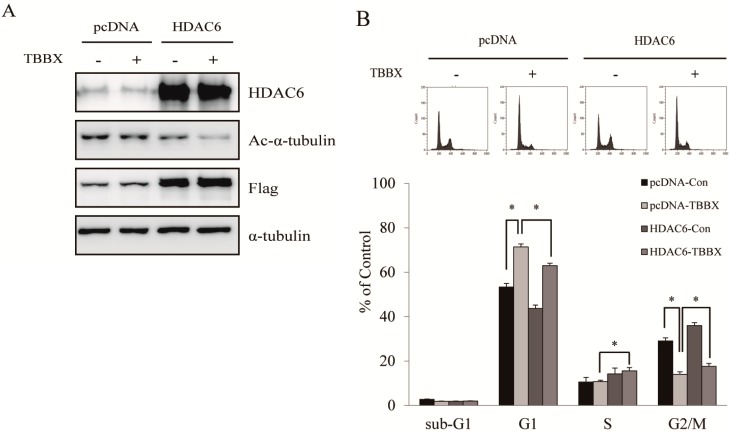
Ectopic expression of HDAC6 rescued TBBX-induced G1 growth arrest in H1299 lung cancer cell. H1299 cells were transient transfected HDAC6-overexpressed plasmid or vector only as described in Materials and Methods. Afterward, cells were treated with 10 μM TBBX for 24 h. (**A**) Cells were then harvested and Western blotting analyses performed by anti-HDAC6, acetyl-α-tubulin, flag-tag and anti-α-tubulin. Data shown are representative of at least three independent experiments. (**B**) After TBBX treatment, cell cycle analyses were done as described in Materials and Methods. Data were the mean ± S.D. of triplicate samples. Significant difference was observed from the control group (* *p* < 0.05).

HDAC6-regulated cell proliferation via modulation of Hsp90 acetylation has been well characterized [16,47,48]. Acetylation Hsp90 by HDAC6 leads to disrupt Hsp90 chaperone function and promote the degradation of client proteins [14,15,16]. Furthermore, increasing of Hsp90 acetylation has been demonstrated to mediate the interruption of Hsp90 with cyclin D and CDK4 association [33,42,49,50,51,52]. The effects of TBBX in HDAC6 activity were explored to ascertain the role of HDAC6-Hsp90 signaling pathway in TBBX-treated cells. HDAC6 activity was not inhibited by TBBX treatment in cell-free system (Figure 7A) whereas endogenous HDAC6 activity was repressed in TBBX-treated cells (Figure 7B). Down-regulation of HDAC6 protein level in a dose-dependent mode was also observed in TBBX-treated cells (Figure 7C). Meanwhile, HDAC6 specific substrate, acetyl-tubulin, was similarly accumulated in a dose-dependent mode after TBBX treatment. Immune-precipitation of Hsp90 was performed to detect hyper-acetylation of Hsp90 by Western blot to establish the role of HDAC6 in Hsp90 chaperone function disruption. Figure 6B showed that the acetyl-lysine of Hsp90 was increased after TBBX stimulation (Figure 6B). The relative densities of acetyl-lysine in TBBX-incubated cells were increased about 2.2-fold compared with control group (Figure 6B). Thereafter, H1299 cells were transfected with flag-tag HDAC6 plasmid before TBBX treatment and cell cycle distribution was analyzed to comprehend the relationships between TBBX-mediated G1 growth arrest and HDAC6 signaling disruption. In Figure 8A, overexpressed flag-tag HDAC6 was attenuated TBBX-induced acetyl-tubulin expression. Meanwhile, TBBX-induced G1 growth arrest was rescued by the ectopic HDAC6 expression (Figure 8B). Accordingly, the results implicated that TBBX-induced G1 growth arrest was through the down-regulation of HDAC6-Hsp90 signaling pathway.

### 2.6. Discussion

In this study, the semisynthesized molecule from osthole, NBM-T-BBX-OS01 (TBBX), induced dose-dependent G1 arrest in H1299 cells (Figure 2). TBBX-induced G1 arrest was through down-regulated cyclin D1, CDK2 and CDK4 expression (Figure 3). It was also shown that CDK inhibitors, p21^Waf1/Cip1^ rather than p27^Kip1^, was up-regulated through transcriptional regulation in TBBX-treated H1299 cells (Figure 4). Down-regulation of cyclin D1 and CDK4 expression by TBBX resulted from the disruption of Hsp90 with cyclin D1 and CDK4 complex and led to proteasome degradation (Figure 6A). The results also revealed that TBBX inhibited HDAC6 activity caused by HDAC6 protein down-regulation (Figure 7). Then, down-regulation of HDAC6 expression by TBBX induced Hsp90 hyper-acetylation (Figure 6B). Furthermore, ectopic expression HDAC6 was shown to rescue TBBX-induced G1 growth arrest (Figure 8).

Cell cycle regulatory proteins are abnormal expression and leads to cell cycle dysregulation in tumor cells [36]. Moreover, D- and E-type cyclins bind to their partner protein CDKs and regulate cell cycle transition from the G1 to S phase. Cells enter S phase are administered by the activities of cyclin D/CDK4/6 and cyclin E/CDK2 complexes. During S phase, CDK2 is continually activated and decreased in activity when cells pass into mitosis thereafter [36]. Mutations in cyclin D1 and CDKs are one of the most common genetic alternations present in human cancer [37,38]. Ectopic expression of cyclin D protein has been found in invasive NSCLC [37]. Additionally, high cyclin D1 expression has been established to associate with shorter survival and poor prognosis in NSCLC patients [39]. Thus, down-regulation of cyclins and CDKs expression or interruption cyclins and CDKs interaction might provide the target for cancer therapy. In the study, the anti-lung cancer activity of semisynthesized compound, TBBX, from osthole was examined (Figure 1). At first, the growth inhibitory activity of TBBX in lung cancer cells was inspected. The data exposed that the cell growth of lung cancer cells was inhibited in a dose-dependent mode by TBBX treatment (Figure 2A). Besides, the results also exhibited that anti-proliferative activity of TBBX was more effective than SAHA (Figure 2A). To verify the cytotoxic effects in TBBX-treated cells through cell cycle distribution, H1299 lung cancer cells was chosen as a study model. G1 cell cycle arrest was observed in TBBX-treated H1299 cells (Figure 2B). The G1 phase-accumulated cells were increased about 30% after 10 μM of TBBX treatment. The down-regulation of cyclin D1, CDK2 and CDK4 expression and up-regulation of cyclin E expression by TBBX treatment were also observed (Figure 3). Cyclin E/CDK2 is the key enzyme for regulating G1-S phase transition. Over-expression cyclin E has been observed in many cancers [53]. Induction of G1 growth arrest through down-regulation of cyclin E expression has been investigated in many anti-cancer compounds. However, DNA damage reagents induce the transcription of E2F1 gene through ATM/ATR signaling pathway resulting in cyclin E up-regulation [54]. Therefore, TBBX inducing cyclin E expression to mediate other mechanisms such as DNA damage induction were speculated.

CDK inhibitors (CDKIs) have been established to associate with CDKs monomer or cyclin/CDK complexes resulting in inhibiting complex activities and cell arrest [36]. Up-regulation of CDKIs expression through transcriptional activation or increase in CDKs protein stability has been shown the anti-cancer properties [55,56]. In this study, CDKI, p21^Waf1/Cip1^ instead of p27^Kip1^, was increased in a dose-dependent manner (Figure 4A). Up-regulation of p21^Waf1/Cip1^ protein expression was directed from transcriptional activation by TBBX treatment (Figure 4B). The results implied that TBBX-induced G1 growth arrest might be through down-regulation of cyclin D1, CDK2 and CDK4 expression in H1299 lung cancer cells. Meanwhile, TBBX also induced CDK inhibitor p21^Waf1/Cip1^ gene expression leading to blockade cyclins/CDKs activity.

Chromatin modification is a fundamental mechanism of regulating gene expression. It has been identified that histone acetylation of the p27^Kip1^ promoter is an important pathway to govern p27^Kip1^ gene expression [57]. Moreover, histone acetyl-transferase p300 and PCAF also acetylate p27^Kip1^ protein at K100 residue and promote p27^Kip1^ degradation [58]. In our study, down-regulation p27^Kip1^ expressions were observed in TBBX-treated H1299 cells (Figure 4A). We speculated that TBBX might also induce p27^Kip1^ protein acetylation and promote degradation. Besides, inhibition of Hsp90 expression has been demonstrated to promote p27^Kip1^ degradation through destabilizing Cks, an essential component of SCF-Skp2 ubiquitin ligase complex that targets p27^Kip1^ [59]. It could not be excluded that down-regulation p27^Kip1^ expression via TBBX might be through the regulation of ubiquitin-proteasomal system. It is important to verify the role of TBBX in p27^Kip1^ protein regulation in future study.

CDK inhibitor p21^Waf1/Cip1^ is both regulated by p53-dependent and -independent pathways [60]. Tumor suppressor protein p53 transcriptionally up-regulates p21^Waf1/Cip1^ gene expression and leads to growth arrest [46]. However, TBBX-induced p21^Waf1/Cip1^ expression was through p53-independent pathway due to H1299 cells are p53-null type lung cancer cell line [61]. Epigenetic regulation in cancer cells by histone acetylation which is controlled by histone acetyltransferase (HAT)/histone deacetylase (HDAC) affects the transcription by relaxing the chromatin structure and accesses the transcription factors to entry target DNA leading to regulate gene expression [11]. Inhibition of class I HDAC activity via HDAC inhibitor, such as SAHA or valproic acid, has been demonstrated to up-regulate p21^Waf1/Cip1^ gene expression [24,25,26]. To further verify whether TBBX-induced growth arrest was through class I HDAC-mediated p21^Waf1/Cip1^ signaling, class I HDAC activity assay by cell-free system was performed. The data revealed that class I HDAC signaling was not inhibited by TBBX (Figure 5). Meanwhile, TBBX-induced p21^Waf1/Cip1^ expression was not mediated by class I HDAC signaling either. Further verification of the up-regulation mechanism of p21^Waf1/Cip1^ expression via TBBX is vital to the medical application.

Recent studies indicate that the disruption of Hsp90 chaperone function by hyper-acetylation results in client protein degradation via proteasome system leading to growth inhibition in cancer cells [62]. The client proteins of Hsp90 possess important roles in regulation of cell cycle such as cyclins and CDKs [63]. To understand whether down-regulation of cyclin D1 and CDK4 via TBBX was through proteasome degradation, proteasome inhibitor MG132 was used. The outcomes revealed that down-regulation of CDK4 and cyclin D1 expression via TBBX was rescued by MG132 treatment (Figure 6A). To demonstrate whether down-regulation of cyclin D1 and CDK4 via TBBX was through the regulation of Hsp90 chaperone function, H1299 cells were treated with TBBX and immuno-precipitation analyses to detect Hsp90 and cyclin D1 or CDK4 association were performed. Both the bound proteins of cyclin D1 and CDK4 with Hsp90 was decrease about 40% after TBBX treatment (Figure 7B). Meanwhile, Hsp90 hyper-acetylation was increased about two-fold in TBBX-treated H1299 cells. The results suggested that down-regulation of cyclin D1 and CDK4 expression via TBBX might through disruption of Hsp90 chaperone function via hyper-acetylation. Though cyclin D1 is not a confirmed Hsp90 client protein, our immuno-precipitation analysis also observed TBBX disrupted cyclin D1/Hsp90 interaction (Figure 6B). Since cyclin D1/CDK4 complex controls G1 cell cycle progression, the cyclin D1/Hsp90 interaction might come from cyclin D1/CDK4/Hsp90 complex association. Furthermore, hyper-acetylation of Hsp90 has been well known to disrupt Hsp90 chaperone function via HDAC inhibitor (trichostatin A, TSA) leading to cyclin D1 degradation. Trichostatin A induces cyclin D1 nuclear export and promotes cyclin D1 ubiquitylation and proteasomal degradation via up-regulates Skp2 expression, a component of SCF complex [64]. Pre-treatment with proteasome inhibitor also rescued TBBX-down-regulated cyclin D1 expression (Figure 6A). Down-regulation of cyclin D1 expression via TBBX was similar with TSA. It is interesting to further investigate the molecular mechanism of TBBX regulates cyclin D1 expression.

In addition to histone acetylation, non-histone protein acetylation status also controls many important cell functions [12,13,14,17]. HDAC6, a member of class IIb HDAC, possess the ability to catalyze the removal of acetyl groups from substrates other than histones. HDAC6 has been well known as the deacetylase of -tubulin, Hsp90 and cortactin involving in tumorigenesis [30]. Furthermore, HDAC6-regulated Hsp90 hyper-acetylation shows to induce the dissociation of client proteins and followed by client protein degradation [15,65]. To investigate whether TBBX-induced hyper-acetylation of Hsp90 was mediated by HDAC6 signaling pathway, cell-free system of HDAC6 activity analysis was performed. The results revealed that HDAC6 activity was not directly inhibited by TBBX treatment (Figure 7A). Interestingly, endogenous HDAC6 activity was inhibited in a dose-dependent manner via TBBX treatment (Figure 7B). Furthermore, the protein level of HDAC6 was down-regulated in a dose-dependent mode after TBBX treatment (Figure 7C). Meanwhile, the specific substrate of HDAC6, hyper-acetylation of α-tubulin, was increased in TBBX-treated cells (Figure 7C). Conclusively, inhibition of HDAC6 activity by TBBX was through down-regulation of HDAC6 protein expression and TBBX-induced G1 arrest might be through HDAC6-mediated signaling. To further understand the role of HDAC6 in TBBX induced G1 arrest, ectopic HDAC6 expression was performed. As shown in Figure 8A, up-regulation of acetyl-tubulin via TBBX was rescued after overexpression HDAC6 via transient transfection. The G1-accumulated cells via TBBX treatment was also attenuated in ectopic HDAC6 cells (Figure 8B). TBBX-induced G1 population cells were rescued about 10% after HDAC6 overexpression. Accordingly, the results suggested that TBBX-induced G1 growth arrest was through HDAC6 signaling down-regulation. Down-regulation of HDAC6 expression via TBBX induced Hsp90 hyper-acetylation and followed by disassociation with cyclin D1 and CDK4. This disassociation might promote CDK4 and cyclin D1 degradation by proteasome-dependent pathway in H1299 cells. The discoveries might provide the new strategy for lung cancer treatment.

## 3. Experimental Section

### 3.1. Chemicals and Reagents

NBM-T-BBX-OS01 (TBBX) was provided from NatureWise Biotech & Medicals Corporation (Taipei, Taiwan). The purities (>99%) were confirmed by ^1^H-NMR and HPLC analyses. Anti-cyclin D1, E, CDK2, CDK4, p21^Waf1/Cip1^, p27^Kip1^, HDAC6, acetyl lysine and anti-acetyl-α-tubulin antibodies were purchased from Cell Signaling (Beverly, MA, USA). Anti-β-actin antibody and MG132 were obtained from Sigma-Aldrich (St. Louis, MO, USA). Anti-Hsp90 antibody and protein A/G plus agarose were acquired from Santa Cruz Biotechnology (Santa Cruz, CA, USA). HDAC6 activity assay kit was gotten from Biomol/Enzo Life Science International, Inc. (Plymouth Meeting, PA, USA).

### 3.2. Cell Culture and Cytotoxicity Assay

NSCLC H1299, H460, A549, and H1155 cell lines were obtained from American Type Culture Collection (Manassas, VA, USA). All of cell lines were cultured in RPMI-1640 (Hyclone Laboratories, Logan, UT, USA) supplemented with 5% fetal bovine serum and maintained at 37 °C in a humidified atmosphere at 95% air and 5% CO_2_. All cells (1 × 10^4^/well) were seeded in 96-well plates and incubated for 24 h. Cells were then treated with various dosage of TBBX for 24 h. At the end of incubation, cell viability was determined by MTT assay.

### 3.3. Cell Cycle Analysis

H1299 cells were plated and then synchronized for 24 h. After synchronization, the media were changed to complementary media and TBBX (0, 2.5, 5, 7.5 and 10 μM) was added for 24 h. Cells were then harvested and stained with propidium iodide (50 μg∙mL^−1^) (Sigma Chemical, St. Louis, MO, USA). DNA contents were measured using a FACScan laser flow cytometer analysis system (Beckman Coulter, Fullerton, CA, USA). 

### 3.4. Western Blot Analysis

Western blot analysis was performed as described previously [60]. Briefly, control and TBBX-treated cell lysates were collected via protein extraction buffer. Cell lysates were quantitated, electrophoresed, and transferred to Immobilon polyvinylidene difluoride membranes (Millipore Co., Billerica, MA, USA). The membranes were blocked with blocking buffer and incubated with the indicated antibodies. The signals were detected by chemiluminescence (ECL kit, Amersham Pharmacia Biotech, Piscataway, NJ, USA) and were quantitated by a UVP BioSpectrum Imaging System ChemiDoc-It2 810 (UVP LLC, Upland, CA, USA). The expression of β-actin was used as the internal control.

### 3.5. Immuno-Precipitation

Immuno-precipitation experiments were performed as previously described [60]. Briefly, cell lysates (1 mg in 200 L of protein extraction buffer) were incubated with an anti-Hsp90 antibody and protein A/G plus agarose at 4 °C for 18 h. The immune-complexes were washed via immuno-precipitation buffer and resuspended in 25 L of protein loading buffer. The immune-complexes were then performed by Western blot analyses. The blots were incubated with anti-ubiquitin (Santa Cruz Biotechnology, Santa Cruz, CA, USA), anti-cyclin D1, anti-CDK4 or anti-acetyl lysine primary antibodies. For internal control, blots were then stripped and reprobed with anti-Hsp90 antibody. 

### 3.6. Reverse Transcription Polymerase Chain Reaction (RT-PCR)

RT-PCR was performed as described previously [60]. Briefly, total RNA was isolated by RNA mini kit (Qiagen, Taipei, Taiwan). cDNAs were prepared using a high capacity cDNA reverse transcription kit (Invitrogen, Taipei, Taiwan) following the manufacturer’s protocol. p21^Waf1/Cip1^ and GAPDH were amplified by a thermal cycler in a 25 L of the PCR reaction mixture. p21^Waf1/Cip1^, forward primer 5'-GGCGCCATGTCAGAACCGGCTG-3' and reverse primer 5'-ACCCAGCGGACAAGTGGGGAGG-3', yielded an amplicon of 851 bp while the GAPDH, forward primer 5'-TGAAGGTCGGAGTCAACGGGTGAGTT-3' and reverse primer 5'-CATGTAGACCCCTTGAAGAGG-3', yielded an amplicon of 983 bp. The amplification conditions were as following: an initial denaturation at 95 °C for 5 min, 26 cycles of amplification for p21^Waf1/Cip1^ (95 °C for 50 s, 60 °C for 45 s, and 72 °C for 60 s) or 30 cycles of amplification for GAPDH (94 °C for 50 s, 60 °C for 45 s, and 72 °C for 120 s), and a final extension step at 72 °C for 10 min. The PCR products were separated by 1.8% agarose gel and then visualized by SYBR Safe (Life Technologies, Taiwan) staining. Gene expressions were quantitated using an UVP BioSpectrum Imaging System ChemiDoc-It2 810 (UVP LLC).

### 3.7. HDAC Activity Detection

Class I HDAC and HDAC6 activity was measured by the *Fluor-de-Lys*^®^ HDAC (BML-AK500) and HDAC6 (BML-AK516) fluorometric drug discovery kit (Biomol/Enzo Life Science International, Inc., Plymouth Meeting, PA, USA) according to the manufacturer’s protocol. Briefly, TBBX-treated H1299 cell lysates were incubated with the assay buffer containing HDAC6 assay substrate and then terminated with developer solution. The fluorescence was measured at an excitation wavelength of 360 nm and emission wavelength of 460 nm by Tecab Infinite^®^ 200 PRO (Tecan Group Ltd., Männedorf, Switzerland). The direct inhibitory effects of TBBX on class I HDAC and HDAC6 protein activity were examined using HeLa cell nuclear extracts (BML-AK500) and human HDAC6 recombinant protein (BML-AK516) and followed the manufacturer’s protocol. We also analyzed trichostatin A (TSA) as positive controls.

### 3.8. Ectopic Overexpression of HDAC6

C-terminal FLAG-tagged cDNA of HDAC6 (kindly provided by Dr. Eric Verdin, Gladstone Institute of Virology and Immunology, University of California, San Francisco) was cloned into pcDNA3.1 plasmid vector (Invitrogen). H1299 cells were cultured to 70% confluence and then transfected with control vector or Flag-tag HDAC6 plasmid using PolyJet^TM^ In Vitro DNA Transfection Reagent (SignaGen Laboratories, Ijamsville, MD, USA) according to the manufacturer’s instructions. After transfection, cellular levels of the HDAC6, Flag-tag and acetyl-tubulin proteins were checked by immunoblotting.

### 3.9. Statistical Analysis

The results were expressed as mean ± SD calculated from the specified numbers of determination. One-way ANOVA was used to compare individual datum with control value. A probability of *p* < 0.05 was taken as denoting a significant difference from control data.
